# A new icriodontid conodont cluster with specific mesowear supports an alternative apparatus motion model for Icriodontidae

**DOI:** 10.1080/14772019.2017.1354090

**Published:** 2017-08-15

**Authors:** Thomas J. Suttner, Erika Kido, Antonino Briguglio

**Affiliations:** a University of Graz, Institute for Earth Sciences, Heinrichstrasse 26, 8010Graz, Austria; b Geological-Palaeontological Department, Natural History Museum Vienna, Burgring 7, 1010Vienna, Austria; c Faculty of Science, Universiti Brunei Darussalam, Jalan Tungku Link, GadongBE1410, Brunei Darussalam

**Keywords:** *Caudicriodus woschmidti*, conodont cluster, apparatus reconstruction, mesowear, mastication model

## Abstract

Increasing numbers of conodont discoveries with soft tissue preservation, natural assemblages and fused clusters of the hard tissue have strengthened the hypothesis regarding the function and mechanism of the conodont feeding apparatus. Exceptional fossil preservation serves as a solid basis for modern reconstructions of the conodont apparatus illustrating the complex interplay of the single apparatus elements. Reliable published models concern the ozarkodinid apparatus of Pennsylvanian and Early Triassic conodonts. Recognition of microwear and mammal-like occlusion, especially of platform elements belonging to individuals of the genus *Idiognathodus*, allows rotational closure to be interpreted as the crushing mechanism of ozarkodinid platform (P1) elements. Here we describe a new icriodontid conodont cluster of *Caudicriodus woschmidti* that consists of one pair of icriodontan (I) and 10 pairs of coniform (C1–5) elements, with I elements being preserved in interlocking position. The special kind of element arrangement within the fused cluster provides new insights into icriodontid apparatus reconstruction and notation of elements. However, orientation of coniform elements is limited to a certain degree by possible preservational bias. Four possible apparatus models are introduced and discussed. Recognition of specific wear on denticle tips of one of the icriodontan elements forms the basis for an alternative hypothesis of apparatus motion. Analysis of tip wear suggests a horizontal, slightly elliptical motion of opposed, antagonistically operating I elements. This is supported by similar tip wear from much better preserved, but isolated, elements of Middle Devonian icriodontids. More detailed interpretation of the masticatory movement will allow enhanced understanding of anatomical specifications, diet and palaeobiology of different euconodont groups.

## Introduction

Fused clusters of icriodontid conodonts have been known since the late 1960s. The first publication discussed the apparatus of the Late Devonian species *Icriodus alternatus* (Lange 1968). This paper concluded that about 30 coniform elements belonging to the form taxon *Acodina*, together with one pair of icriodontan elements, could represent the apparatus of one individual. Concerns regarding the absolute number of acodinan elements were raised by Lange (1968) because the conodont elements were clustered within a coprolite. In the early 1980s, more than 850 conodont clusters of *Icriodus expansus* from Late Devonian deposits of the Canning Basin in Western Australia were analysed and described by Nicoll (1982). He concluded that the apparatus consisted of one pair of icriodontan (I) elements and more than 140 cone elements. Nicoll discriminated seven element types and changed the common opinion of a bimembrate (Klapper & Philip 1971) or trimembrate (Nicoll 1977) into a septimembrate icriodontid apparatus model (Nicoll 1982). Since then, the notation and number of elements included within the individual apparatus has changed, especially among Early Devonian icriodontids. However, these models of the apparatus have been reconstructed on statistical analysis of isolated elements, which are supposed to belong to *Caudicriodus woschmidti* (Serpagli 1983) and *Cypricriodus hesperius* (Simpson 1998; Murphy *et al*. 2016). Serpagli (1983) introduced an icriodontid apparatus model that includes a set of ramiform elements.

The conodont cluster of *Caudicriodus woschmidti* described here provides new insights into apparatus composition and notation of elements. The architecture of the cluster is very similar to the apparatus composition described by Nicoll (1982), except that coniform elements, which have prominent shoulder spurs, are lacking. It differs from the reconstructions of Serpagli (1983) in having no ramiform or denticulate elements preserved. Therefore, the notation of coniform elements by Nicoll (1982) is modified here.

Although clusters of icriodontan elements in interlocking position were found prior to our discovery, distinctive tip wear was not recognized. Weddige (1990) attempted to classify wear of isolated conodont specimens, resulting in the identification of *Occlusio*, *Depressio* and *Duplicatio* pathologies for the genera *Polygnathus* and *Icriodus*. Based on pathologies seen on the oral surface of conodont elements, Weddige (1990) hypothesized a permanent see-saw movement of antagonistically working platform element pairs. Alternative models of the motion of the icriodontid apparatus do not exist. Thus, recognition of denticle tip wear on one of the icriodontan elements of the *Caudicriodus* cluster provides a unique opportunity to reconsider the icriodontid apparatus motion model. Analysis of mesowear (Purnell & Jones 2012) on the fused cluster is supported by measurements of denticle tip wear on isolated, but much better preserved, Middle Devonian icriodontan elements.

## Material and methods

Extraction of conodonts followed the standard chemical methods for phosphatic microfossils (Jeppsson & Anehus 1995). For dissolution of conodont-bearing rocks (marl and limestone), we used 5% formic acid. Sodium polytungstate (density 2.79 g/cm^3^) was used for heavy liquid separation (Mitchell & Heckert 2010).

Scanning electron microscope (SEM) images of conodonts were produced using a Zeiss DSM 982 Gemini electron microscope (Institute for Earth Sciences, University of Graz). Conodonts were coated with gold/palladium alloy for 10 minutes.

For three-dimensional reconstruction and discrimination of single apparatus elements, computer microtomography (Micro-CT) was used for the icriodontid conodont cluster. The specimen was scanned for *c*. 5 hours at 80 kV (source voltage) and 100 uA (source current) with an image rotation step of 0.2600 degrees using a SkyScan 1173 (Department of Palaeontology, University of Vienna).

The material figured herein was collected from two localities. The conodont cluster of *Caudicriodus woschmidti* was obtained from bed Ki/4/2a (*hesperius* Biozone, Lochkovian) of the ‘Kottwitz’ quarry near the village of Kirchfidisch, southern Burgenland, Austria (Suttner 2009a). Two isolated icriodontan elements of *Icriodus* aff. *michiganus* and *Icriodus* sp. came from sample BL-12-29c (*kockelianus* Biozone, Eifelian), Blankenheim Syncline, Eifel, Germany (Königshof *et al*. 2016).

The conodont cluster of *Caudicriodus woschmidti* is stored in the micropalaeontological collection of the Geological-Palaeontological Department, Natural History Museum Vienna (Austria) under the repository number NHMW 2011/0374/0001.

## Geological setting

**Figure 1.**
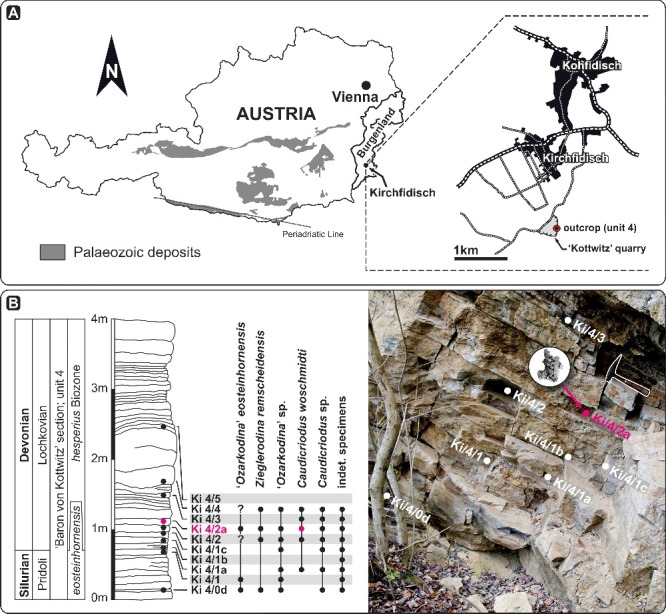
Locality map and section log from the ‘Kottwitz’ quarry (southern Burgenland, Austria), where the *Caudicriodus woschmidti* conodont cluster was found.

The icriodontid conodont cluster was found in Unit 4 of the ‘Kottwitz’ quarry near Kirchfidisch (Fig. 1), which represents one of few localities in southern Burgenland (Austria) where Silurian to Devonian rocks are exposed. Other outcrops are found near the villages of Hannersdorf, Punitz and Sulz (Pollak 1962; Schönlaub 1984, 1994, 2000). Additionally, the sequence is documented from subsurface drilling cores in the Styrian Basin near the villages of Arnwiesen and Blumau (Ebner 1988; Flügel 1988).

At the ‘Kottwitz’ quarry, the latest Silurian (Pridolian) and Early Devonian (Lochkovian) sequence is about 40 metres thick and consists of phyllitic shale, calcareous marls, laminated limestone, dolomitic limestone and dolostone (Suttner & Lukeneder 2004; Suttner 2009a). The interval yielding the fused cluster of *Caudicriodus* comprises well-bedded, laminated argillaceous and silty limestones. Beds are 7 to 25 cm thick and yield a low-diversity invertebrate fauna including brachiopods, ostracods and crinoid ossicles. Other specimens of fused coniform elements (icriodontids) and ramiform elements (ozarkodinids) were found in beds Ki/4/1c and Ki/4/2a (Suttner 2009b, c).

## Systematic palaeontology

### 

#### General remarks

Although we use the term Conodonta of Eichenberg (1930), the subdivisions of order (Paraconodontida excluded) and family as introduced by Sweet & Donoghue (2001, fig. 6) are followed here. A slightly modified notation of Nicoll (1982) for coniform elements of the *Caudicriodus* apparatus is used. This Early Devonian species did not possess coniform elements with prominent shoulder spurs (Ce element of Nicoll 1982) and therefore the apparatus reconstruction of Nicoll (1982) cannot be applied entirely, although all other element types are recognized. In order to keep the relation to Late Devonian icriodontids, we retain the notation of ‘C’ for ‘coniform’ but use numbers instead of letters for subdivision of elements. Our revised notation based on Nicoll (1982) is as follows: Ca = C3; Cb = C5; Cc = C1; Cd = C4; Ce = not observed; Cf = C2a–f; I = I.

Because *in vivo* orientation of icriodontid apparatus elements is unknown, we apply conventional terminology and orientation here for description of elements and indication of relative disposition in proposed models. Terms used follow the suggestions of Purnell *et al*. (2000, pp. 117, 119, table 2).

Phylum **Chordata** Bateson, 1886

Class **Conodonta** Eichenberg, 1930 *sensu* Sweet & Donoghue, 2001

Order **Prioniodontida** Dzik, 1976

Family **Icriodontidae** Müller & Müller, 1957

Genus ***Caudicriodus*** Bultynck, 1976

#### Type species

*Icriodus woschmidti* Ziegler, 1960; lower Lochkovian; Untenrüden, Rhenish Slate Mountains, Germany.

#### Remarks

The generic diagnosis follows Bultynck (1976). The outline of the widely opened basal cavity (termed platform in earlier publications, but not homologous to the platform in polygnathids, for example) is identical in shape to that of other species of *Icriodus*. Features which discriminate *Caudicriodus* from *Icriodus* are the lateral process (denticulate or adenticulate) that extends posterior of the cusp at a specific angle, and a spur developed between the cusp and ‘posterior’-most transverse denticle row on the ‘inner’ side of the element.

Bultynck (1976) included the following species within *Caudicriodus*: *Caudicriodus woschmidti*, *C. postwoschmidti*, *C. angustoides*, *C. curvicauda*, *C. celtibericus* and *C. sigmoidalis*. Later, Drygant (2010) included four additional species, *Caudicriodus hesperius*, *C. ruthmawsonae*, *C. transiens* and *C. serus*, and Drygant & Szaniawski (2012) described *Caudicriodus schoenlaubi*. More recently, Murphy *et al*. (2016) excluded *Caudicriodus hesperius*, placing it in their new genus *Cypricriodus*.

***Caudicriodus woschmidti*** (Ziegler, 1960)

(Figs 2, 4)

1960 *Icriodus woschmidti* Ziegler: 185, pl. 15, figs 16–18, 20–22.

1962 *Icriodus woschmidti* Ziegler; Jentzsch: 967, pl. 1, figs 17–23.

1964 *Icriodus woschmidti* Ziegler; Walliser: 38, pl. 9, fig. 22, pl. 11, figs 14–22.

1969 *Icriodus woschmidti transiens* Carls & Gandl: 174, pl. 15, figs 1–7.

1969 *Icriodus woschmidti* Ziegler; Klapper: 10, pl. 2, figs 3–5.

1975 *Icriodus woschmidti woschmidti* Ziegler; Carls: 410, pl. 2, figs 19–21.

1976 *Caudicriodus woschmidti* (Ziegler); Bultynck: 21, figs 1, 3–4 [cum syn.].

1977 *Caudicriodus woschmidti* (Ziegler); Bultynck: pl. 39, fig. 10, pl. 40, fig. 24.

1977 *Icriodus woschmidti woschmidti* Ziegler; Chatterton & Perry: 793, pl. 3, figs 18–22.

1980 *Icriodus woschmidti woschmidti* Ziegler; Jaeger & Schönlaub: pl. 4, figs 4–5/16, 6/16.

1980 *Icriodus woschmidti woschmidti* Ziegler; Pickett: 70, fig 3B–D.

1980 *Icriodus woschmidti* Ziegler; Serpagli & Mastandrea: 39, figs 2–4.

1981 *Caudicriodus woschmidti woschmidti* (Ziegler); Norris & Uyeno: pl. 5, figs 10–17.

1981 *Icriodus woschmidti* Ziegler; Wang: 77, pl. 1, figs 22–25.

1983 *Icriodus woschmidti* Ziegler; Broadhead & McComb: 153, figs 2E, 3H–J.

1983 *Icriodus woschmidti woschmidti* Ziegler; Serpagli: 155, figs 2, 5–7.

1986 *Caudicriodus woschmidti woschmidti* (Ziegler); Borremans & Bultynck: 52, pl. 1, figs 1–9.

1988 *Icriodus woschmidti woschmidti* Ziegler; Denkler & Harris: B8, pl. 1, figs A, B.

1990 *Icriodus woschmidti woschmidti* Ziegler; Olivieri & Serpagli: 63, pl. 1, figs 12–14.

1990 aff. *Icriodus* cf. *postwoschmidti* Mashkova; Weyant & Morzadec: 752, pl. 1, figs 1, 3–5.

1994 *Icriodus woschmidti woschmidti* Ziegler; Valenzuela-Ríos: 87, pl. 8, figs 14, 15, 28.

1995 *Icriodus woschmidti woschmidti* Ziegler; Luppold: pl. 2, fig. 11.

1998 *Icriodus woschmidti woschmidti* Ziegler; Çapkinoğlu & Bektaş: 167, pl. 5, figs 10, 11.

1999 *Caudicriodus woschmidti* (Ziegler); Benfrika: 318, pl. 1, fig. 10.

2002 *Icriodus woschmidti woschmidti* Ziegler; García-López *et al*.: pl. 1, figs 5–7.

2003 *Caudicriodus woschmidti woschmidti* (Ziegler); Bultynck: pl. 1, figs 1–3.

2005 *Icriodus woschmidti woschmidti* Ziegler; Corradini *et al*.: fig. 5e.

2009a *Icriodus woschmidti woschmidti* Ziegler; Suttner: 77, pl. 1, figs 1–6.

2010 *Caudicriodus woschmidti* (Ziegler); Drygant: 57, pl. 2, figs 3, 6–13.

2012 *Caudicriodus woschmidti* (Ziegler); Drygant & Szaniawski: 846, figs 9B, 10C, D.

#### Material

NHMW 2011/0374/0001, single conodont cluster including 10 pairs of coniform and both I elements. Additional icriodontan elements from the same locality were described by Suttner (2009a).

#### Description

**Figure 2.**
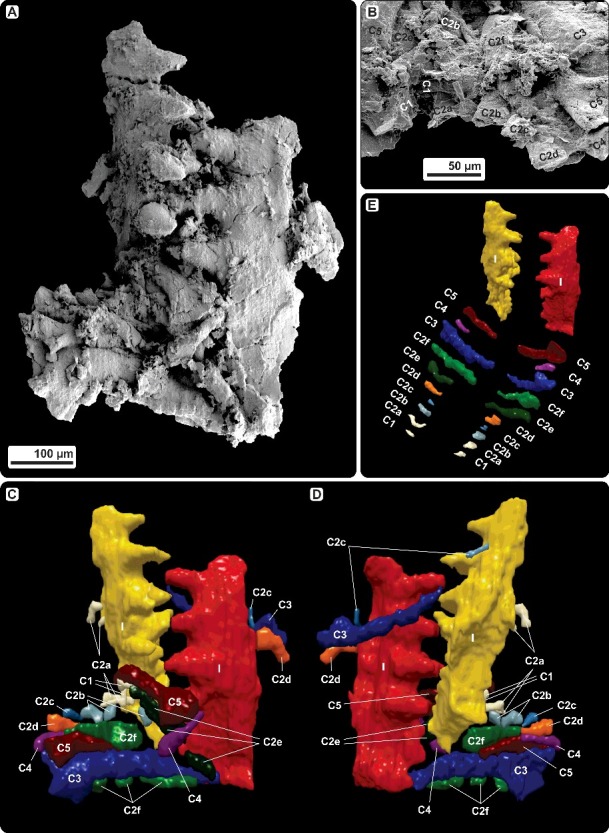
Conodont cluster of *Caudicriodus woschmidti*, Early Devonian, southern Burgenland, Austria; Ki/4/2a-1, NHMW 2011/0374/0001. **A,** SEM scan of the conodont cluster. **B,** detailed view of the coniform elements (C1–C5) close to the dextral I element. **C, D,** computer microtomography-based three-dimensional reconstruction with identification of all elements. **E,** hypothetical arrangement of all elements preserved within the fused conodont cluster.

The icriodontid conodont cluster consists of crown tissue only and includes one pair of I elements and 20 coniform elements, which can be distinguished in 10 pairs (C1–C5). No basal plate is preserved. Although the cluster shows numerous micro-fractures on the surface of I elements with some coniform elements being broken in two or more pieces (still attached in the cluster) or having lost their tips, it can be reconstructed based on SEM and micro-CT analysis (Fig. 2).

Icriodontan elements are preserved with the oral side opposing each other in an interlocking position. Lateral walls of the basal cavity, especially in the ‘posterior’ part of either element, are adpressed and show strong fractures. Therefore, the lower margin of the basal cavity is very irregular, not reflecting the original outline. However, the basal cavity of this species is widest below the cusp. The initial part of the ‘anterior’ portion of both I elements is broken off. Additionally, the ‘posterior’-most portion of the lateral process is broken too. Four transverse denticle rows are bar-like (denticles are connected by high ridges; Fig. 4B) with deep interspacing on the rather low spindle. Some of the lateral row denticles show strong fractures. No surface ornamentation is observed.

Coniform elements are clustered in bidirectional orientation around the ‘posterior’ part of the icriodontan elements (‘inner’ side). Few elements are found on the ‘outer’ side of the sinistral I element which indicate post-mortem distortion of the original orientation of the coniform assemblage. Basically, two sets of different-shaped pairs of small (C1, C2a, C2b, C2c, C2d, C4) and large (C2e, C2f, C3, C5) coniform elements are observed. All of these are adenticulate.

C1 elements are small, gracile coniform elements with a recurved cusp and striate surface ornamentation. The cusp and basal outline are elliptical in cross section (‘posterior’ margin more convex than ‘anterior’ margin) with sharp margins that represent costae.

C2 elements (C2a–C2f) differ in size but all possess a circular outline of the basal margin. All are erect or slightly recurved and show a striate surface ornament where preserved. Generally, neither costae nor keels are developed. Some of elements have the base fractured and therefore the basal margin appears elliptically compressed. C2a elements are broken into two parts: base with major part of cusp and tip of cusp preserved close to each other. The tip of the cusp seems elliptical in cross section (‘posterior’ margin more convex than ‘anterior’ margin) with rather sharp margins. This differs somewhat from other C2 elements which have a cusp with a rather round cross section. C2e and C2f elements are larger than other C2 elements, comparable in size to C3 and C5 elements. However, C2 elements can be discriminated easily by having a circular basal outline and a more slender shape in general. Because of recrystallization, surface ornamentation of C2e and C2f is difficult to ascertain.

The largest pair of coniform elements is identified as C3. Both cones have a widely excavated base with an irregular, flared outline. Elements have a keel extending from the base of the cone to the base of the cusp. The angle between the ‘posterior’ lower part and the ‘posterior’ margin of the cusp is about 97°. The angle between the lower and ‘anterior’ margin of the element is about 55°, slightly curved in the lower one-fifth, continuing rather straight towards the tip of the cusp. No surface ornament is observed.

C4 elements are erect and seem symmetrical with an oval outline of the base. Although it is rather small, one element of the C4 pair is preserved with the same orientation between two large coniform elements, close to the ‘anterior’ margins of C3 and C5.

C5 elements are about half the size of C3 elements, with an erect cusp and a wide, probably oval to circular basal margin. Although the base appears rather conical, the original outline and shape is unknown because of post-mortem deformation. Neither costae nor keels are observed.

#### Remarks

**Figure 3.**
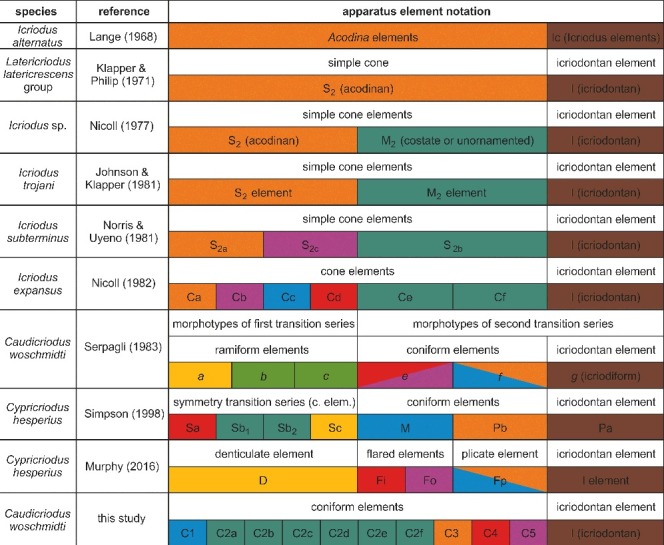
Chronological listing of notation history for icriodontid apparatus elements. Morphologically similar coniform element types and the icriodontan element evaluated for this study are highlighted in different colours or shades.

A chronological summary of the icriodontid element notation (Fig. 3) shows that a bimembrate nature of the apparatus was suggested by Lange (1968) based on the first finding of clusters of *Icriodus alternatus*. A few years later, coniform elements were termed S_2_ (acodinan) elements by Klapper & Philip (1971). Although previously speculated upon by Klapper & Ziegler (1975), Nicoll (1977) was the first to propose a trimembrate apparatus by including an additional type of coniform element (M_2_ element). Further evidence to support this model came from statistical analysis of the apparatus reconstruction of *Icriodus trojani* by Johnson & Klapper (1981). In the same year, Norris & Uyeno (1981) introduced three coniform types (S_2a_, S_2b_ and S_2c_) for the apparatus of *Icriodus subterminus*, of which their S_2a_ element equates with the classically known S_2_ element, and their S_2b_ element with the M_2_ element of Nicoll (1977). Nicoll (1982) set a milestone with his publication on the analysis of hundreds of fused clusters of *Icriodus expansus* from the Canning Basin in which he revised the apparatus architecture and notation of icriodontid conodonts. His reconstruction includes one pair of opposed platform elements (I elements) and other associated coniform elements (Ca, Cb, Cc, Cd, Ce and Cf elements). No ramiform elements are included within this apparatus. However, the large number of coniform elements counted in single clusters led to the conclusion that these were arranged serially within the apparatus of one individual. Like *Icriodus expansus*, the fused cluster of *Caudicriodus woschmidti* did not preserve ramiform elements. The latter elements are part of the apparatus reconstruction suggested by Serpagli (1983). His analysis of disarticulated elements of *Caudicriodus woschmidti* from the Early Devonian of southern Sardinia (Italy) resulted in an apparatus that included ramiform (a, b and c), coniform (e and f) and icriodiform (g) elements. These formed two transitional series, each consisting of three morphotypes (a, b, c and e, f, g). This hypothesis followed the analysis of *Cypricriodus hesperius* from the Silurian to Devonian of north Queensland, Australia, by Simpson (1998), who proposed an apparatus that contained variably ornamented coniform elements (Sa, Sb_1_, Sb_2_ and Sc elements), M elements, Pb elements and Pa elements. In his model, S elements represent a symmetry transition series. Originally introduced for the skeletal apparatus of *Oulodus* by Sweet & Schönlaub (1975), this notation scheme was used by Simpson (1998) for documenting the analogous relationship regarding the position occupied by elements in different euconodont apparatuses. The most recent study of *Cypricriodus hesperius* by Murphy *et al*. (2016) suggested a new apparatus structure followed by introduction of a new element notation based on statistical analysis of isolated elements. These authors discriminated five elements, including three flared elements: one with plication (Fp), a second with the ‘inner’ wall of the base straighter than the ‘outer’ wall (Fi), and a third with the ‘outer’ wall straighter than the ‘inner’ wall (Fo). The fourth coniform element is denticulate (D) and the fifth is represented by the icriodontan element (I). However, apparatus architecture and notation schemes for Early Devonian icriodontids are based exclusively on statistical analysis of isolated elements, which is expected to suffer a higher bias error compared with analysis of fused conodont clusters (see discussion of ‘bias and biology’ by Purnell & Donoghue 2005). Therefore, these are not applied here.

## Results

### Element notation

The skeletal apparatus of *Caudicriodus woschmidti* consists of 10 pairs of coniform (C1, C2a–f, C3, C4 and C5) and one pair of icriodontan (I) elements. Because of the specific arrangement of elements within the cluster, elements are considered to belong to one individual only. The *in vivo* orientation and position occupied by coniform elements and their relative position to the I element pair remain uncertain.

### Recognition of meso- and microwear

**Figure 4.**
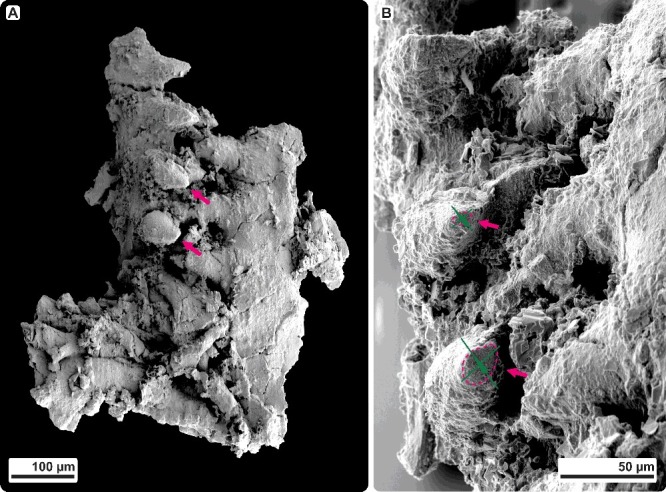
**A,** denticle tip wear of the dextral I element of *Caudicriodus woschmidti*; Early Devonian, southern Burgenland, Austria; Ki/4/2a-1, NHMW 2011/0374/0001. **B,** detailed view of oral surface of the dextral I element with extent and orientation of tip wear indicated by dotted line and arrow head.

Occlusion of icriodontid elements resulted in specifically directed mesowear of denticle tips. Within the *Caudicriodus woschmidti* cluster, mesowear is documented only from denticles 1 and 2 of the ‘inner’ lateral denticle row (dextral I element). Smoothly polished facets of rather elliptical outline are inclined in a more or less ‘anterior’ direction (Fig. 4A, B, indicated by arrows). Because of poor preservation, no further meso- or microwear could be observed with certainty. In order to verify whether tip wear recognized on the dextral I element of the *Caudicriodus* cluster is due to occlusal stress and not merely post-depositional breakage, a search has been made for additional collections of much better preserved, but isolated icriodontan elements.

**Figure 5.**
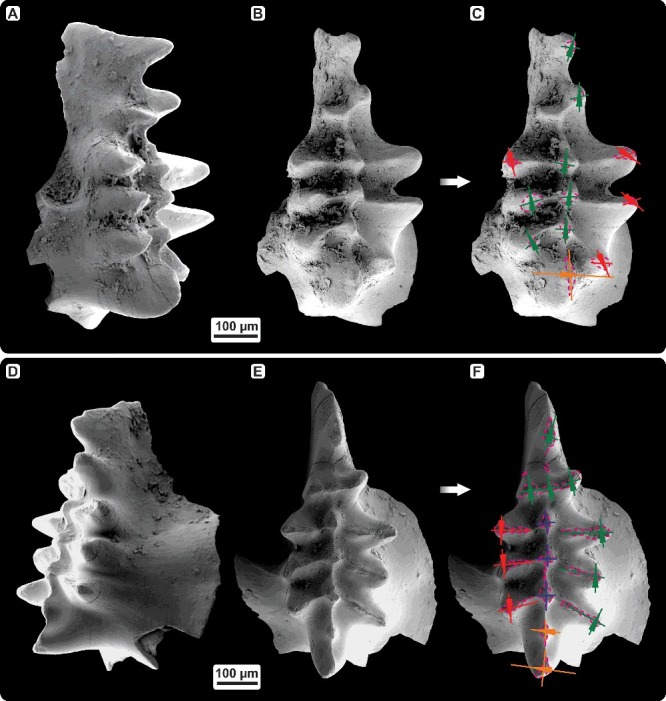
Denticle tip wear of icriodontid I elements. **A–C,** *Icriodus* aff. *michiganus*, dextral I element, lateral and oral view; Middle Devonian, Eifel, Germany; sample BL-12-29c-9. **D–F,** *Icriodus* sp., dextral I element, lateral and oral view; Middle Devonian, Eifel, Germany; sample BL-12-29c-3. Extent and orientation of tip wear are indicated by dotted lines and arrowheads.

**Figure 6.**
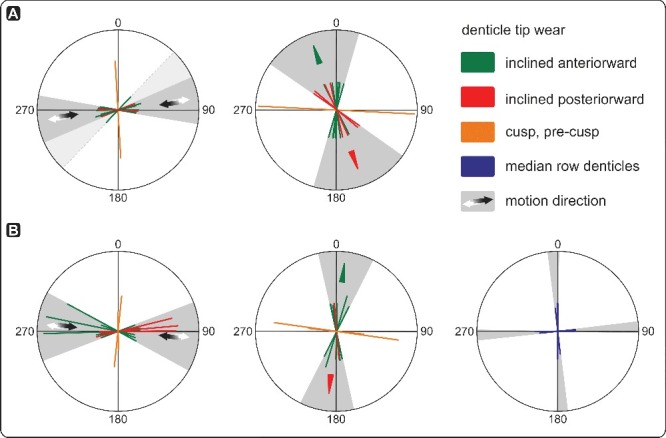
Diagrams illustrating orientation and direction of denticle tip wear. **A,** *Icriodus* aff. *michiganus*; left-side illustration shows the orientation of the inclined facet plane, right-side illustration the direction of vertically inclined facet; Middle Devonian, Eifel, Germany; sample BL-12-29c-9. B, *Icriodus* sp. left-side illustration shows the orientation of the inclined facet plane, middle the direction of the vertically inclined facet, and right the orientation and direction of the facet plane of median row denticles; Middle Devonian, Eifel, Germany; sample BL-12-29c-3).

We found several late Eifelian icriodontan elements (Eifel area, Germany) possessing similar wear. Tip wear of two elements is analysed in detail and illustrated in Figures 5 and 6. The first icriodontan element of *Icriodus* aff. *michiganus* (Figs 5A–C, 6A; Supplemental Fig. 1) shows that tip wear of three median row denticles is located on the ‘inner’ –‘posterior’ denticle quarter and inclined in an ‘anterior’ direction. Tip wear of lateral row denticles is documented in opposite denticle quarters (‘outer’–‘posterior’ vs ‘inner’–‘anterior’) with an inverted inclination angle. The largest denticle on the oral surface (= cusp), shows wear along the ‘inner’ side, which is inclined in an ‘outer’ direction nearly perpendicular to the direction of wear measured from other denticles (Fig. 6A). There is a range of variation of tip wear observed within one element. Some denticles are damaged more strongly compared to others possessing steeply inclined, rather ovate to elliptical outlined wear facets that reach deeply down to the denticle base (Fig. 5A–C: ‘inner’ lateral row denticle 2 and median row denticle 2). Other tips show a less inclined crescent-shaped facet. Three denticles clearly have layered microstructure exposed on the somewhat blunted tips (Fig. 5A–C: ‘inner’ lateral row denticle 3 and ‘outer’ lateral row denticles 2 and 3, counted from the ‘posterior’ towards the ‘anterior’).

For comparison, the crown tissue of a second icriodontan specimen from the same locality is illustrated in Figures 5D–F and 6B. It shows a more strongly and rather roughly damaged oral surface. Tips of median row denticles possess planar wear facets without a specific inclination direction (Figs 5D–F, 6B). However, spalling occurs on median row denticles. It is documented on the ‘inner’, ‘posterior’ and ‘outer’ side of denticle 1, and the ‘inner’–‘posterior’ and ‘posterior’ side of denticles 2 and 3, respectively. Although a major part of the ‘inner’ lateral row denticle 2 is broken (Fig. 5D–F), direction of wear can be reconstructed based on the remaining damage of the transverse ridge extending from the lateral row denticles towards the base of middle row denticles. The ‘posterior’-most three ‘inner’ lateral row denticles possess facets which are inclined ‘posteriorly’. Those of the ‘outer’ lateral row denticles and the entire fourth denticle row show opposite inclination (the process of facet formation is illustrated for the fourth transverse denticle row in Supplemental Video 1). Some of the transverse ridges also show spalling (‘inner’ lateral row denticle 1 and ‘outer’ lateral row denticle 2 on the ‘posterior’ side of the transverse ridge, and ‘outer’ lateral row denticle 3 on either side). Similar to the other specimen, wear of the cusp (and here the pre-cusp too) is nearly perpendicular to tip wear of the other denticles. Spalling is observed within the ‘posterior’ –‘outer’ quarter of cusp and pre-cusp. Although both specimens are dextral elements, the inclination of wear of the cusp runs in the opposite direction.

Microwear is observed only on a few isolated late Eifelian coniform elements from the conodont collection of the Eifel area (Germany). Well-preserved specimens show a smoothly polished tip of the otherwise striate cusp.

## Discussion

A significant difference exists between the *Caudicriodus* cluster and those published by Nicoll (1982). Most coniform elements of the specimen shown here are attached nearly perpendicularly to the I element pair, possessing a more or less bidirectional orientation (Fig. 2). Compared to this, coniform elements of the *Icriodus expansus* clusters are oriented either chaotically around the icriodontan elements (Nicoll 1982, fig. 4.Aa) or show a rather parallel unidirectional arrangement alongside the faecal pellet (Nicoll 1982, fig. 11). Such specific orientation and the quantity of 10 element-pairs (it is unclear whether additional elements are missing) suggest that the conodont cluster represents skeletal remnants of one individual only. Furthermore, the presence of coniform elements in pairs suggests bilaterally symmetrical apparatus architecture. This is supported by microwear of some coniform elements published by Nicoll (1982, fig. 10.Ic, Mc), where sharp lateral margins of the cones’ cusps are produced by polishing away the striate micro-ornament on the lateral edges (of either one or both sides) of the ‘anterior’ and/or ‘posterior’ surface. Other cusps of coniform elements show removed micro-ornament only in the tip-region of the ‘posterior’ surface (Nicoll 1982, fig. 10.Rc). Such microwear is considered to be the result of ‘tooth-to-tooth’ contact of opposing interdigitating coniform elements.

However, a discrepancy can be recognized regarding the absolute orientation of the elements when comparing the *in vivo* orientation proposed for the apparatuses of ozarkodinids (Aldridge *et al*. 1987, 1993; Purnell 1993, 1995; Purnell & Donoghue 1997; Purnell *et al*. 2000) and prioniodontids (Gabbott *et al*. 1995; Freedman 1999; Purnell *et al*. 2000). All of these have the denticle tips of ramiform elements oriented in a dorsal direction with the ‘posterior’ part of the platform elements oriented in the same direction (originally based on the apparatus model deduced from individuals of *Clydagnathus* by Aldridge *et al*. 1987). In the *Caudicriodus* cluster, almost all denticle tips of coniform elements point in an opposite direction relative to the orientation of the ‘posterior’ part of the icriodontan element. This kind of preservational bias allows only hypothetical reconstructions. Based on the arrangement and orientation of elements and without having evidence of *in vivo* orientation of icriodontids based on natural assemblages with soft tissue preservation, four possible models of the icriodontid apparatus architecture are introduced and discussed below.

**Figure 7.**
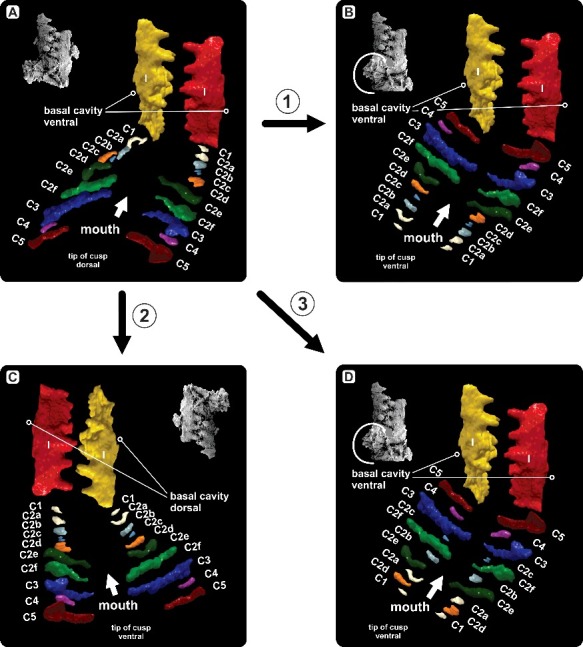
Hypothetical apparatus reconstruction deduced from the element arrangement within the *Caudicriodus woschmidti* conodont cluster. **A,** Model 1 with tips of coniform elements pointing dorsally and ‘posterior’ part of icriodontan elements oriented ventrally. **B,** Model 2 with tips of coniform elements and ‘posterior’ part of icriodontan elements oriented ventrally. **C,** Model 3 with tips of coniform elements pointing ventrally and ‘posterior’ part of icriodontan elements oriented dorsally. **D,** Model 4 with tips of coniform elements and ‘posterior’ part of icriodontan elements oriented ventrally. Coniform elements are arranged in multiple rows.

In the first model (Fig. 7A), the original post-mortem orientation of the majority of coniform elements relative to the icriodontan pair is illustrated. Except for four coniform elements (C2a, C2c, C2d and C3), all are clustering near the ‘posterior’ part of the interlocked I element pair. Most of the coniform elements are oriented with the ‘posterior’ margin of the cusp pointing ‘posteriorly’. A few elements are oriented in the opposite direction (with the ‘anterior’ margin of the cusp pointing ‘posteriorly’: each of C2a, C2c and C3), or with one of lateral margins, base or tip ‘posteriorly’ (each of C1, C2a, C2b and C4). Apparently, this is due to post-mortem displacement. In the apparatus reconstruction, these elements are reoriented such that the ‘posterior’ margin points in a ‘posterior’ direction with the tip of the cusp dorsal, a reconstruction resembling the *in vivo* orientation in the sense of Aldridge *et al*. (1987) for ozarkodinids. The apparatus would have most of the small coniform elements (C1–C2d, except for C4) in a position close to the icriodontan element pair. Large coniform elements (C2e–C3 and C5) would cover a position in front of the small coniform group. Although orientation of coniform elements in this model (with the tip of the cusp dorsal) accords with the *in vivo* orientation suggested for ramiform elements of ozarkodinids, the orientation of icriodontan elements (‘posterior’ part in a ventral direction) contradicts the published *in vivo* orientation of most models since Aldridge *et al*. (1987). Surprisingly, it follows the conventional orientation suggested for isolated icriodontan elements (Branson & Mehl 1938).

In the second model (Fig. 7B), conventional orientation of icriodontan elements is retained because no proof of opposite orientation is known for icriodontid conodonts. Here, the coniform part is reoriented with the tip of the cusp pointing ventrally. The model is based on the assumption that collapse of the head was followed by post-mortem displacement of the coniform elements – at that time still connected. Such displacement could have resulted in rotation and partial disintegration of the coniform apparatus part. In this case, small elements are located in front of the large coniform elements.

In the third model (Fig. 7C) orientation of the icriodontan element pair follows the *in vivo* orientation of platform elements suggested for ozarkodinids (*sensu* Purnell *et al*. 2000). Here, coniform element tips would point ventrally and large elements would cover a position in front of small elements.

The fourth model (Fig. 7D) hypothesizes an absolutely different apparatus architecture consisting of multiple rows of coniform elements. Generally, orientation of coniform and icriodontan elements follows the second model (Fig. 7B), but here the coniform part of the apparatus is divided into two rows of alternating elements. The lower row consists of small and the upper row of large elements. The model is based on the evidence that one of the C4 elements is preserved between C3 and C5. Additionally, most of the small elements (C1–C2d) are preserved close to each other within the cluster and are located between the icriodontan pair and the group of large coniform elements (C2e–C5). Such specific sorting of coniform elements points to a neighbouring position covered by the groups of small and large coniform elements in the original apparatus configuration.

### Existing apparatus motion models

Earlier models of the euconodont apparatus largely resulted in the reconstruction of commonly known disarticulated elements. The discovery of conodont clusters and natural assemblages with soft tissue preservation and the observation of meso- and microwear on the surfaces of the crown tissue opened the path to new directions in conodont research towards a better understanding of conodont palaeobiology (Nicoll 1982, 1984, 1987, 1995; Briggs *et al*. 1983; Aldridge *et al*. 1987, 1993, 1995; Sweet 1988; Gabbott *et al*. 1995; Purnell 1995, 2001; Donoghue & Purnell 1999a; Donoghue *et al*. 2000, 2008; Purnell *et al*. 2000; Sweet & Donoghue 2001; Zhuravlev 2007; Jones *et al*. 2012; Purnell & Jones 2012; Martínez-Pérez *et al*. 2014a, b, 2016).

**Figure 8.**
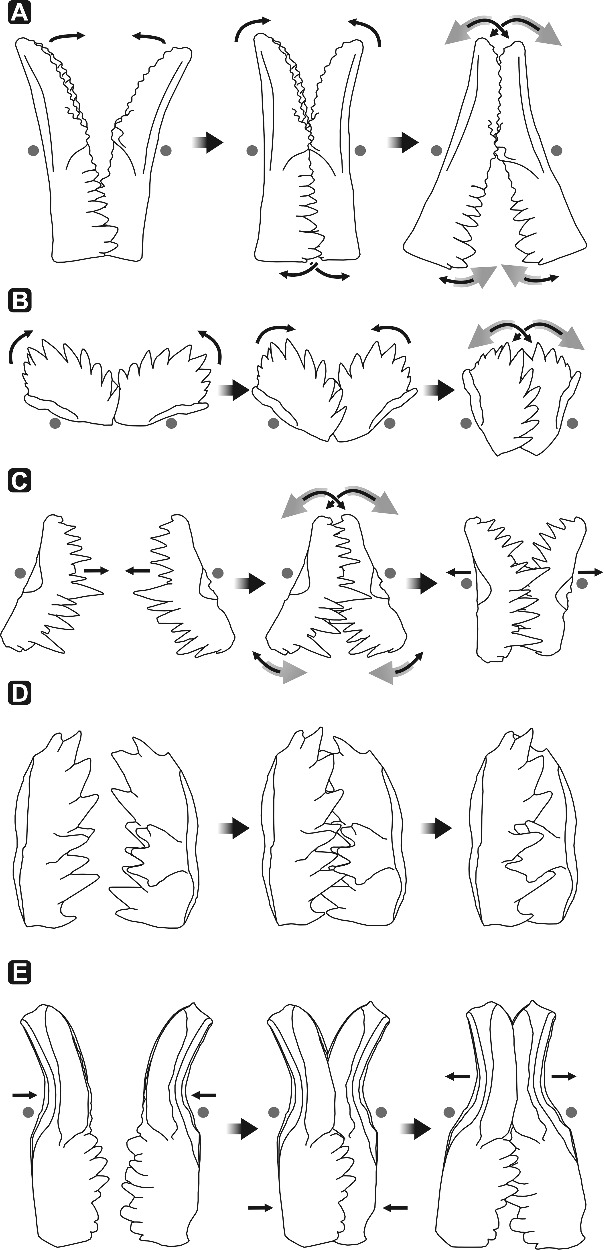
Motion of P1 elements of ozarkodinid apparatuses summarized from the literature. **A,** *Idiognathodus* (Pennsylvanian); **B,** *Novispathodus* (Early Triassic); **C,** *Wurmiella excavata* (Silurian); **D,** *Pseudofurnishius murcianus* (Middle–Late Triassic); **E,** *Polygnathus xylus xylus* (Middle Devonian). Grey dots mark the pivot point; black arrows indicate the direction of occlusion and interlocking of P1 elements, grey arrows its reversal.

The type model for ozarkodinid apparatuses (Purnell & Donoghue 1997, 1998) is based on the analysis of wear and surface damage of articulated platform element pairs from natural assemblages of *Idiognathodus*. It concludes that opposing platform elements were located close to each other in a slightly offset position, performing a rotational movement, with the pivot point on the ventral part of the element where the platform joins the free blade (Fig. 8A). This movement resulted in a complex interlocking occlusion of the oral surface of P1 elements (Donoghue & Purnell 1999b).

A similar kind of platform element motion was suggested for Early Triassic individuals of the genus *Novispathodus* (Fig. 8B). In this reconstruction, P elements persistently operate in synchronous rotational movement by shearing the lateral blade surfaces against each other, while ramiform elements (M and S types) act independently to grasp hard tissue (Goudemand *et al*. 2011). Coordination of such apparatus motion requires a complex arrangement of muscle tissue.

Jones *et al*. (2012) suggested that in addition to rotational occlusion, elements must have separated during the occlusal cycle (Fig. 8C), because of smoothly polished wear on either side of lateral surfaces of the elements (occlusal and non-occlusal sides). Based on sharpness analysis they concluded that the sharper dorsal edges of cusp and denticles indicate a dorsal rotation direction of the primary power stroke of the platform elements. The authors pointed out that separation of platform elements during the occlusal cycle also allowed food to move between elements, which would otherwise be difficult. Studies on functional morphology and element kinematics by Jones *et al*. (2012) were performed on natural assemblages of the Silurian species *Wurmiella excavata*.

Martínez-Pérez *et al*. (2014a) provided a slightly different model for platform element motion after studying two fused clusters from Slovenia (Krivic & Stojanovič 1978) and additional disarticulated platform elements from Spain (Plasencia 2009) of the Middle–Late Triassic conodont *Pseudofurnishius murcianus*. Similar to the model for *Wurmiella excavata* of Jones *et al*. (2012), elements are considered to separate completely during each occlusal cycle, moving more or less orthogonally to the oral surface against each other for the next power stroke. Occlusion was refined by interdigitation of platform denticles. In this model, rotational occlusion is not a major part of the occlusal cycle (Fig. 8D). However, it is considered possible when P1 elements are interlocked. Smooth polishing, chipping and spalling are observed on either lateral surface of the cusp and denticles (more weakly developed on the non-occlusal side), demonstrating that occlusion was not always precise (Martínez-Pérez *et al*. 2014a).

Another apparatus motion model based on the study of clusters of *Polygnathus xylus xylus* (Fig. 8E) was introduced by Martínez-Pérez *et al*. (2016). Originally the material was published by Nicoll (1984), who illustrated an apparatus model with ‘anterior’–‘posterior’ axis of P1 and P2 elements in rostro-caudal orientation. Martínez-Pérez *et al*. (2016) revised the orientation of platform elements according to the suggestions of Purnell *et al*. (2000) and proposed a new motion model for P1 elements of *Polygnathus*. In this model, opposing P1 elements are brought together bilaterally with the blade of the left element behind the right element. Blades act as guides, aligning elements while platforms approach each other. Once elements are in interlocking position, a short rotational movement follows from ventral to dorsal along the elements’ curvature. Because of imprecise occlusion of the carina and the platform troughs in the dorsal region of the platform, rotational occlusion is considered to stop in the middle part of the platform, not contacting dorsal-most regions of the element pair.

### Alternative model for icriodontid apparatus motion

**Figure 9.**
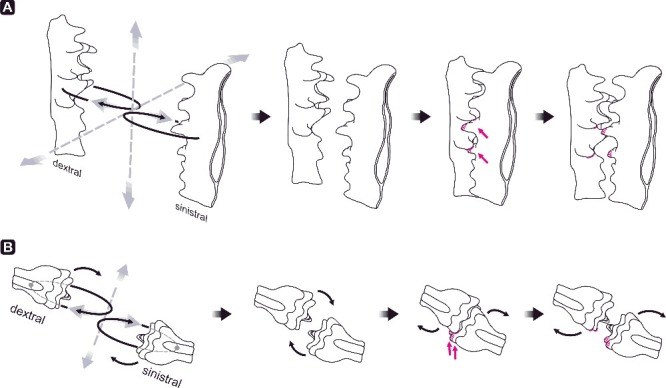
Model of masticatory motion of icriodontid I elements. **A,** oblique lateral view; **B,** ‘anterior’ view.

Direction of breakage and stress on elements (Purnell 1995; Zhuravlev 2007; Jones *et al*. 2012; Purnell & Jones 2012; Martínez-Pérez *et al*. 2014a, 2016), supported by analogous pathodynamic mechanisms of tooth wear in dental sciences (e.g. Grippo *et al*. 2004; Fondriest & Ralgrodski 2012), results in a slightly different model of apparatus motion for icriodontid conodonts. Compared to other models for ozarkodinid P1 elements (Jones *et al*. 2012; Martínez-Pérez *et al*. 2014a, 2016), oral surfaces of opposed icriodontid I elements approach each other with a slightly elliptical rather than directly orthogonal motion (Fig. 9; Supplemental Video 2). We agree with Jones *et al*. (2012) that occlusion was not always precise depending on the morphology of the oral surface and related to denticle guidance during the element interlocking process. However, rotational movement as suggested for ozarkodinids is not part of the occlusal cycle in icriodontids. In this respect, no specific meso- and/or microwear is observed.

Because of poor preservation, tip wear of coniform elements is difficult to ascertain from the *Caudicriodus* cluster. However, some coniform elements possess narrow ‘posterior’ keels or costae on either lateral side, which implies coniform guidance. Additional hints on apparatus function and motion can be inferred from isolated coniform elements from the collection containing icriodontan elements of the genus *Icriodus* (Fig. 5). Specific occlusion patterns such as smoothing of striate micro-ornament on the ‘posterior’ surface of the tip of the cusp or the lateral margin of either the ‘posterior’ and/or ‘anterior’ surface can be documented. Such wear is characteristic for element-element attrition (see Jones *et al*. 2012). Distinctively different element types show specific attrition patterns; thus, it is assumed that coniform elements operated – probably arranged in multiple rows – against each other, organized in a bilaterally symmetrical disposition during the occlusal cycle (compare tip wear published by Nicoll 1982, fig. 10.Ic, Mc, Rc). However, architecture and function of the coniform part of the icriodontid apparatus are not understood well enough to provide a model of apparatus motion illustrating how coniform and icriodontan elements worked together within one individual apparatus. Such studies will be the topic of future research.

## Conclusions

The fused conodont cluster of *Caudicriodus woschmidti* provides new insights into the apparatus structure of Early Devonian icriodontid conodonts. The apparatus consists of 11 element pairs (10 pairs of coniform elements and one pair of icriodontan elements). Specific post-mortem arrangement of most coniform elements suggests that all elements are skeletal remnants of one individual only. The icriodontid element notation provided by Nicoll (1982) for *Icriodus expansus* can be adopted in part. One of the coniform element types of Nicoll (1982, Ce element) was not observed. Contrary to earlier reconstructions of *Caudicriodus woschmidti* (Serpagli 1983), no ramiform elements are preserved within the described cluster. However, a preservational bias is evident which restricts our conclusions regarding the absolute number of coniform elements and its orientation relative to the I element pair. With this in mind, we discuss four hypothetical models on the apparatus architecture of *Caudicriodus woschmidti*.

Distinctive tip wear is observed on one of the icriodontan elements. Together with more significant results from meso- and microwear analyses of additional Middle Devonian icriodontan conodont material from the Eifel area (Germany), a new model for icriodontid apparatus motion is suggested. The occlusal cycle consists of a slightly elliptical rather than straight orthogonal motion of opposed I elements when approaching each other for the interlocking phase. Rotational movement in interlocking position is not considered for this euconodont group. We recognize that neither does element-element attrition always affect all denticles on the oral side, nor is tip wear present within the same area of each denticle. The investigated material shows that density, inclination and orientation of tip wear are related mainly to individual denticle size, growth form and the relative position of denticles on oral sides of opposed elements during the interlocking phase. Compared to this, the occlusal cycle of coniform elements based on microwear is less well understood. Wear on the cusp is located in specific areas of different element types and related to removed striate micro-ornament. Two types of microwear are observed: (1) polished tip of the cusp (‘anterior’ and/or ‘posterior’ side), and (2) polished and sharpened lateral margins (left and/or right margin of ‘anterior’ and/or ‘posterior’ side).

The many unsolved questions regarding the orientation and position of coniform elements preclude complete reconstruction of the icriodontid apparatus motion model. Illustration of the interaction of coniform and icriodontan elements during the occlusal cycle will be issue for future study based on additional conodont clusters and natural assemblages with soft tissue preservation.

## Supplementary Material

Suttner_etal_SI_Video2.mpg

Suttner_etal_SI_Video1.mpg

Suttner_etal_SI_Fig1.tif
